# Depression among people with epilepsy in Northwest Ethiopia: a cross-sectional institution based study

**DOI:** 10.1186/s13104-015-1515-z

**Published:** 2015-10-19

**Authors:** Berhanu Boru Bifftu, Berihun Assefa Dachew, Bewket Tadesse Tiruneh, Nigusie Birhan Tebeje

**Affiliations:** Department of Nursing, University of Gondar College of Medicine and Health Science, P.O.Box: 196, Gondar, Ethiopia

**Keywords:** Antiepileptic drugs, Depression, Epilepsy, Perceived stress

## Abstract

**Background:**

Epilepsy is the world’s most common neurological disorder, affecting approximately 50 million people worldwide and contributed to different psychiatric illness. Depression is one of the most frequent co morbid psychiatric disorders that affects the life of the patients’. This study aimed to assess the prevalence of depression and associated factors among epileptic patients attending the outpatient department of the University of Gondar Hospital, Northwest Ethiopia, 2014.

**Methods:**

Institution based quantitative cross—sectional study was conducted among 405 individual with epilepsy. The participants were selected using systematic random sampling technique. Semistructured questionnaires were used to obtain socio-demographic and clinical data. Depression was measured using Beck’s Depression Inventory Binary logistic regression used for analysis.

**Results:**

The estimated, prevalence of depression was found to be 45.2 %. Out of these (29.6 %) were classified as mild, (14.8 %) as moderate and (0.8 %) were severely depressed. A lower educational status was associated with an increased prevalence of depression and the adjusted odds ratio (AOR) for the illiterate [can’t read and write] was 8.32 [95 % Confidence Interval (CI): 4.83, 14.29]. Perceived stress (AOR = 6.21, CI 3.69, 10.44), onset of illness <6 years (AOR = 5.29, CI 4.09, 15.89), seizure frequency of [1–11 per year (AOR = 1.34, CI 1.41, 4.36), ≥1 per month (AOR = 7.83, CI 3.52, 17.40)], poly-pharmacy (AOR = 7.63, CI 2.74, 21.26)] and difficulties of adherence to antiepileptic drugs (AOR = 4.80, CI 2.57, 8.96) were also found to be independently associated with depression.

**Conclusion:**

Overall, the prevalence of depression was found to be high. Lower educational status, early onset of illness, seizure frequency, poly-pharmacy and difficulties of adherence to anti-epileptic drugs (AEDs) were factors statistically associated with depression. Strengthening the educational status of the patients on the effect of early onset of the illness, frequent seizure occurrence and difficulty of adherence to AEDs as a contributing factors for other co-morbid psychiatric disorder are suggested in the clinical care setting.

## Background

Epilepsy is the world’s most common neurological disorder, affecting approximately 50 million people worldwide, and the majority of whom (80–85 %) live in resource-poor countries [1]. The prevalence of epilepsy in African ranges from 2.2 to 58 per 1000 population and constitutes the second or third reason for consultation and hospitalization [2].

In Ethiopia, epilepsy is a huge problem, which affects about 5.2 per 1000 population [3, 4] with an estimation of about 0.5 % of patients with active epilepsy (with seizers in the past 1–2 years). The existing studies also revealed that individuals with epilepsy suffer from a number of social, psychological and physical poor outcomes. For example a study conducted in Ethiopia revealed that 60 % of the study participants faced different problems as a result of their illness such as stigma by 24 %, inability to find a partner by 31 %, educational problems by 17 %, and problems of employment by 9 % of adults [5]. That is why in a recently released Ethiopian Mental Health Strategic Plan, give emphasis to epileptic patients to be seen as one of the special vulnerable groups that require special consideration [6].

According to World Health Organization mental illness is one of the greatest causes of disability universally. Five out of the ten leading causes of disability worldwide can be attributed to psychiatric disorders [7]. Depression is one of the leading causes of disability in the world. It is expected to be the second leading cause of disability worldwide by 2020 [8]. In Ethiopia, mental illness is the leading non-communicable disorder in terms of burden with schizophrenia and depression included in the top ten most burdensome conditions, out-ranking HIV/AIDS [6].

Research evidences had been revealed the multidirectional links between mental and physical health and illness [9]. Thoughts, feelings and health behaviour have a major impact on physical health status. Conversely, physical health status considerably influences mental health and well-being [10, 11].

Depression is one of the co-existing psychiatric disorders with chronic illnesses such as epilepsy [1, 2] that show a bidirectional interaction with epilepsy. For example, a cross sectional study revealed that up to 60 % of epileptic patients developed depression and the presence of depression also increase risk of epilepsy up to 3–7 fold [12]. A comparative study have also shown that the rate of depression in patients with epilepsy is significantly higher than the rate of depression in patients with other chronic diseases such as diabetes or asthma and the general population as well [13].

The presence of depression among epileptic patients can be associated with different psycho-social difficulties on patients life such as poor treatment adherence, poor quality of life, unemployment, lower educational status, increase burden and cost on healthcare services and higher risk for suicide [12–15].

Many studies from the Western world have reported on the prevalence of depression among people with epilepsy and its negative consequences, but few studies have addressed the issue in developing countries. The few studies conducted in Africa reported the widespread existence of depression among epileptic patients. However, the available studies from Western and some African countries demonstrate high prevalence and negative consequences of depression. In Ethiopia, is the authors could find only one previously published study concerning the prevalence of depression among epileptic patients that reported from the Western part of Ethiopia that has a different socio cultural diversity (in Ethiopia within differences ethnic groups there are different language, religion, belief in disease causation) from our study area that may contribute for the variation of illness [16].

Yet, in the Northern part of the country where the culture of the community is fairly different from the Western part of the country (Ethiopia), there is a need to understand the prevalence and its contributing factors. Therefore, looking at the diverse problem related to depression, assessing the prevalence and predictors of depression are important to design more effective treatment programs for the management and preventions of its consequences. Thus, the main purpose of this study was to assess the prevalence and associated factors of depression among people with epilepsy at University of Gondar Hospital, North West Ethiopia, 2014.

## Methods

### Study design

Institution based cross-sectional study design was used.

### Study area and period

The study was conducted at the University of Gondar Hospital (UoGH) from January 01/2014, to February 30/2014. Gondar town is located in North Gondar Administrative Zone of Amhara National, Regional State, 748 km away from the capital city of Ethiopia, Addis Ababa. University of Gondar Hospital serves about 5 million people in the catchment area. It has 400 beds in five different inpatient departments and 14 wards.

### Participants

Participants of this study were individuals with epilepsy receiving follow-up care at the outpatient department of the University of Gondar Hospital. Single population proportion formula (with a 5 % margin of error, 95 % confidence level and 49.3 % proportion) was used to calculate sample size; and it was found to be 422 (including 10 % non response rate). The total number of patients who visited the hospital for the last 12 months were taken from patient records and then the average number of patients per day calculated and then sampling fraction was determined. Finally, participants were selected by systematic random sampling technique every one interval starting from the first patient visiting the epileptic clinic and then continued to the third.

All were consenting individuals with a clinical diagnosis of epilepsy coming for follow up with an age greater than or equal to 18 years were selected. Individuals with epilepsy who were critically ill (can’t give response), unable to speak and hear were excluded from the study.

### Instrument

For the assessment of depression, Beck Depression Inventory (BDI) was used. BDI is one of the most widely used self report measures of depression. It is a reliable and valid measure of depression in a range of cultural groups and has been validated with both psychiatric and non-psychiatric populations in most of the countries including Africa. It consists of 21 items, and each of its items describes a specific symptom of depression. A more recent version of the instrument (BDI-II) was used to correspond to DSM-IV criteria for depression. BDI-II used for screening of recent (during past 2 weeks) depression symptoms in persons with epilepsy. Each statement is scored on a 4-point scale (0–3) and a total score is obtained by summing the ratings for each statement [17]. The prevalence of depression was defined using a cutoff point ≥10 on BDI-II as having depression. A score from 0 to 9 is considered to be within the normal range or asymptomatic; a score of 10–18 indicates mild depression; a score of 19–29 indicates moderate depression and a score of 30 or more severe depression [18, 19].

The questionnaires were translated into Amharic (local language) by an Amharic speaking linguist. The back-translation was performed by mental health specialist into English and then a consensus version was developed in a group discussion by involving the research team. This was compared with the original version, and confirmed to be satisfactory for use. The questionnaires were tested on 21 patients to make it easier for the participants to understand and complete. In this study, BDI-II had an internal consistence of Cronbach’s alpha 0.856 for the total score.

For the assessment of the stress, Perceived Stress Scale (PSS) was used. The PSS is the most widely used psychological tool for measuring the perception of stress. The questions in the PSS asked about the feelings and thoughts of the patients during the past month. Each item is rated on a 5-point scale ranging from never (0) to almost always [4]. Positively worded items are reverse scored, and the ratings are summed, with higher scores indicating more perceived stress. The presence of perceived stress was defined using a cutoff point ≥20 on PSS. PSS had an internal consistence of Cronbach’s alpha for the total score of PSS = 0.793 [20].

Furthermore, we asked one question on antiepileptic medication adherence. The antiepileptic medication adherence question asked about the history of non-adherence with antiepileptic medications with yes/no response. Specifically, we asked “Have you ever discontinued your antiepileptic medication?” Family history of mental illness was assessed by the question, ‘has any member of your family (immediate family- parent, sibling, child) ever been diagnosed with a psychiatric disorder?’ Possible responses to this question were: (a) Yes (b) No. Family social support was assessed by the question ‘How do you rate your social support (immediate family—parent, sibling, child)?’ Possible responses to this question were: (a) Low, (b) Moderate, (c) High and the presence of seizer frequency was assessed by the question, ‘Do you have a seizer for the last 12 months?’ Possible responses to this question were: (a) Yes (b) No. If yes, how many times?

### Data collection and analysis

Data were collected by face-to-face interview using a semi-structured questionnaire with the Amharic version of the socio-demographic, clinical factors, and BDI-II questionnaires.

EPI info version 3.5.3 statistical software and SPSS windows version 16 program were used for analysis. Descriptive statistics (frequencies, tables, percentages, means and standard deviation) were used for the socio-demographic and clinical variables, including individual’s response BDI-II. Binary logistic regression and odds ratio with 95 % confidence interval were used to identify the independently associated factors with depression.

All factors with a p value <0.2 in the bivariate logistic regression were entered into the multivariate model. Statistical significance was accepted at the 5 % level (p < 0.05).

### Ethical consideration

The study proposal was initially approved by the ethical review board of The University of Gondar. A formal letter of permission was obtained from the hospital and submitted to the respective outpatient department. The information about the study was given to the participants. Verbal and then written informed consent was sought for each participant who agreed to participate in the study and full filled the inclusion criteria. Only anonymous data collected in private rooms.

## Results

A total of 405 participants participated in this study with a 96 % response rate. Seventeen questioners were not fulfilled properly or incomplete.

### Socio-demographic and clinical characteristics of participants

The majority of the participants were men 255 (63 %). The mean ages of the participants were 28.75 ± 9.65 years. One hundred and ninety-five (48.1 %) of the participants were primary educated, 372 (91.9 %) were Orthodox and 246 (60.7 %) were married. Three hundred and eighty-four (94.8 %) of the participants were Amhara by Ethnicity. Out of 405 participants, 240 (59.2 %) were self-employed and 213 (52.6 %) were living in urban areas.

And regarding their clinical characteristics, the mean age of disease onset was 20.99 ± 11.16 years. The mean duration of disease was 8.11 ± 6.63. Out of 405 patients, 291 (71.8 %) had seizure frequency range from 1 to 11 times per year. Three hundred and nine (76.3 %) of the participants were treated with one AED (mono-therapy), 132 (32.6 %) had a history of non adherence to AEDs and 18 (4.4 %) had a family history of mental illness (Table 1).

**Table 1:** Distribution of participants by their socio-demographic and clinical characteristics at University of Gondar Hospital, 2014 (n = 405)

Characteristics	Number	Percent
Sex		
Male	255	63
Female	150	37
Age		
18–24	165	40.7
25–34	123	30.4
35–44	87	21.5
≥45	30	7.4
Educational status		
Can’t read and write	117	28.9
Primary	195	48.1
Secondary	51	12.6
College and above	42	10.4
Religion		
Orthodox	372	91.9
Muslim	33	8.1
Marital status		
Married	246	60.7
Single	117	28.9
Divorced/widowed	42	10.3
Ethnicity		
Amhara	384	94.8
Tiger	21	5.2
Employment		
Government	27	6.7
Private	240	59.2
Student	66	17.8
Unemployed	72	16.3
Residence		
Rural	192	47.4
Urban	213	52.6
Social support		
High	249	61.4
Moderate	93	23
Low	63	15.6
Perceived stress		
Low	189	46.7
High	216	53.3
Age at onset of disease in year		
<6	18	4.4
6–11	45	11.1
12–17	111	27.4
18–24	111	27.4
25–34	75	18.5
≥35	45	11.1
Duration of the disease		
<1	39	9.6
2–5	144	35.6
6–10	117	28.9
≥11	105	25.9
Seizure frequency per year		
0	84	20.7
1–11 per year	291	71.8
≥12 per year	30	7.4
Therapy (number of drug)		
Mono-pharmacy	309	76.3
Poly-pharmacy	96	23.7
Difficulties of adherence to AEDs		
Yes	132	32.6
No	273	67.4
Family history of mental illness		
Yes	18	4.4
No	387	95.6

### Prevalence of depression

Overall, the prevalence of depression was found to be 45.2 %. Out of these (29.6 %) mild, (14.8 %) moderate and (0.8 %) were severe depression.

### Factors associated with depression

From the bivariate analysis: age, educational statuses, social support, perceived stress, the onset of the illness, seizure frequency, poly-pharmacy, adherence to AEDs and family history of mental illness were factors associated with depression. On the other hand; sex, religion, marital status, ethnicity, residence, employment and duration of the illness were not associated with depression and excluded from further analysis.

From the multivariate analysis; education status [can’t read and write (AOR = 8.32, CI 4.826, 14.294), primary (AOR = 6.02, CI 3.727, 9.292), secondary (AOR = 4.00, CI 4.102, 8.251)], perceived stress (AOR = 6.21, CI 3.691, 10.442), onset of illness < 6 year (AOR = 5.29, CI 4.086, 15.894), seizure frequency of [1–11 per year (AOR = 1.34, CI 1.414, 4.360), ≥1/month (AOR = 7.83, CI 3.523, 17.400)], polypharmacy (AOR = 7.63, CI 2.741, 21.264) and difficulties of adherence to AEDs (AOR = 4.80, CI 2.568, 8.964) were factors statistically significant with depression at p value <0.05 (Table 2).

**Table 2:** Factors associated with depression (bivariate and multivariate) analysis, at UoGH, 2014

Explanatory variables	Depression Yes N (%) No N (%)	COR (95 % CI)	AOR (95 % CI)	P value
Age				
18–24	66 (40) 99 (60)	0.67 (0.305, 1.455)		
25–34	51 (41.5) 72 (58.5)	0.71 (0.318, 1.577)		
35–44	51 (58.6) 36 (41.4)	1.42 (0.616, 3.259)		
≥45	15 (50) 15 (50)	1		
Educational status				
Can’t read and write	57 (48.7) 60 (51.3)	5.70 (2.233, 14.551)	8.32 (4.826, 14.294)**	<0.001
Primary	90 (46.2) 105 (53.8)	5.14 (2.072, 12.763)	6.02 (3.727, 9.292)**	<0.001
Secondary	30 (58.8) 21 (41.2)	8.57 (3.064, 23.974)	4.00 (4.102, 8.251)**	0.005
College and above	6 (14.3) 36 (85.7)	1	1	
Social support				
High	105 (42.2) 144 (57.8)	1		
Moderate	42 (45.2) 51 (54.8)	1.13 (0.699, 1.825)		
Low	36 (57.142.9) 27 (6.7)	1.83 (1.046, 3.197)		
Perceived stress				
Low	51 (27) 138 (73)	1	1	<0.001
High	132 (61.1) 84 (38.9)	4.25 (2.789, 6.484)	6.21 (3.691, 10.442)**	
Age at onset of the disease in year				
<6	15 (83.3) 3 (0.716.7)	2.50 (0.625, 9.996)	5.29 (4.086, 15.894)**	<0.001
6–11	24 (53.3) 21 (46.7)	0.57 (0.244, 1.341)	0.95 (0.287, 3.169)	0.939
12–17	36 (32.4) 75 (67.6)	0.24 (0.115, 0.501)	0.55 (0.213, 1.413)	0.214
18–24	45 (40.5) 66 (59.5)	0.34 (0.165, 0.705)	0.85 (0.343, 2.153)	0.734
25–34	33 (44) 42 (56)	0.39 (0.182, 0.848)	0.84 (0.330, 2.153)	0.720
≥35	30 (66.7) 15 (33.3)	1	1	
Seizure frequency per year				
0	21 (25) 63 (75)	1	1	1
1–11 per year	147 (50.5) 144 (49.5)	3.06 (1.776, 5.280)	1.34 (1.414, 4.360)**	0.022
≥12per year	15 (50) 15 (50)	3.00 (1.257, 7.157)	7.83 (3.523, 17.400)**	<0.001
Therapy (number of drug)				
Mono-pharmacy	132 (42.7) 177 (57.3)	1	1	<0.001
Poly-pharmacy	51 (53.1) 45 (46.9)	5.34 (1.484, 19.389)	7.63 (2.741, 21.264)**	
Difficulties of adherence to AEDs				
Yes	81 (61.4) 51 (38.6)	2.66 (1.736, 4.083)	4.80 (2.568, 8.964)**	<0.001
No	102 (37.4) 171 (62.6)	1	1	
Family history of mental illness				
Yes	9 (50) 9 (50)	1.22 (0.476, 3.151)		
No	174 (40.3) 258 (59.7)	1		

** Statistically significant using a forward stepwise regression method with p value of Hosmer and Lemeshow goodness fit test 0.344

## Discussion

The aim of this study was to assess the prevalence and associated factors of depression among people with epilepsy at Gondar University Hospital. In this study, the prevalence of depression was found to be 45.2 %. Out of these (29.6 %) mild, (14.8 %) moderate and (0.8 %) were severe depression. This result is similar with the study done in Poland (49.2 %) [21], Mexico (42.7 %) [22], Nigeria (42, 45 %) [23] and Ethiopia (49.3 %) [16]. The results of this study confirm the study done elsewhere as depression is a common co morbid psychiatric disorder among epileptic patients.

This figure (45.2 %) is lower than the study carried out in Korea (62 %) [19], Iraq (51.6 %) [24], Pakistan (60 %) [25], Gaza (63 %) [26], and Nigeria (85.5 %) [27]. This variation may be due to using different diagnostic criteria or different rating scales in diagnosing depression and recruiting epileptic patients with different seizure types, variable frequency and severity, and with different antiepileptic medications. For example study in Pakistan used ICD-10 and their study sample is small (100) as compared to this study which may over estimated the prevalence of depression and in Gaza 64.5 % of study subject have epileptic fits uncontrolled type which contribute for the high depression.

In contrast, it is higher than the study done in USA (9.5) [28], Bosnia (34 %) [29], Thai (20 %) [30] and Egypt (25.5 %) [31]. The possible explanations for the variation may be due to use of different tools, (e.g., Hamilton Rating Scale for Depression (HRSD), Composite International Diagnostic Interview Short Form (CIDI-SF), use of different cutoff points, geographical areas and cultures of the study subject In Egypt out of all the study subject 100 were health individual taken for comparison which may lower the prevalence of depression, in USA they used the Hospital Anxiety and Depression scale was used which has different cutoff point and again the geographical, infrastructure, economic development are different which contribute to lower the symptom of depression and in Bosnia they used prospective.

Regarding the associated factors, those patients who cannot read and write had more than eight times (AOR = 8.32, CI 4.826, 14.294) odds of depression as compared to those patients who had educational status of college and above. Those patients who had educational status of primary (AOR = 6.02, CI 3.727, 9.292) and secondary (AOR = 4.00, CI 4.102, 8.251) had more than six and four times odds of depression as compared to those patients who had educational status of college and above respectively. These results were consistent with the previous study [16, 24, 29]. This is due to the fact that those patients with lower educational status may have poor coping strategies to their illness, which in turn to social isolation, poor adherence to their AEDs, school dropout that impaired their cognition and contributes to poorer psychological adjustment that they face in life.

Those patients with high perceived stress had more than six times (AOR = 6.21, CI 3.691, 10.442) odds of depression as compared to those patients who had low perceived stress. This may be due to the fact that those individual with high perceived stress may have poorer psychological adjustment when they face different stress causing problems such as perceived stigma, unemployment, lower educational status and seizure frequency in their life that may be the cause for the comorbid psychiatric illness. Study shows that the most frequent kinds of stressor seem to be associated with depression were: situations that lower self-esteem, situations where a person is frustrated in the reaching of a certain goal and any single stressor of overwhelming magnitude [32]. A large body of research has also indicated that stress may lead to biochemical changes in the brain, which in turn to induce depression [33]. A cross sectional study reported by S.A. Lee et al. revealed that out of the total 54.7 % of the study explained by variance, perceived stress alone account for 38.8 % for depression and the remaining all other variables account for 15.9 % (that is social support account for 6.8 %, anxiety for 3.9 %, and the remaining three variables (self-efficacy, employment status and age) accounted for 5.2 % [19].

Those patients who had disease onset of less than 6 years had more than five times (AOR = 5.29, CI 4.086, 15.894) odds of depression than those patients who had disease onset at age of 35 years and above. These results were consistent with the previous studies done in Gaza [33]. As different literature revealed epilepsy imposes a substantial burden on the patient their families, the community, and society as a whole [1, 2, 5]. This increased burden is represented in many domains of their life such as physical health [5], psychosocial well-being [1, 3, 16] and economic problem [21, 27]. In our study the study subjects may not have enough coping mechanisms to these different problems because at early stage of life they may not have high level of education and experience that help them to cope up with different cultural belief, stigma and illness that contributed to co morbid psychiatric illnesses. This result indicates that those study participants who had early onset of the disease in their life had more depression compared to those who had late onset of the disease.

Those patients who had seizure frequency of twelve and above had around eight times (AOR = 7.83, CI 3.523, 17.400) odds of depression as compared to those patients who had no seizure frequency and those patients who had seizure frequency of one and above per year were more than one times (AOR = 1.34, CI 1.414, 4.360) more likely to develop depression than those patients who had no seizure frequency. These results were consistent with the previous studies [22, 23, 31, 33]. The possible reason may be since the symptoms and signs of epilepsy are peculiarly unhidden, unpredictable and not easy to understand. This difficulties of understanding where and when the seizure occur may associated with socially unacceptable sign such as loss of stool, urine, foaming from the mouth and tongue biting were evident that lead epileptic patient to develop different psycho-social problem including depression.

Those patients who used poly-pharmacy had more than seven times (AOR = 7.63, CI 2.741, 21.264) odds of depression than those patients who had mono-pharmacy. These results were consistent with the previous studies [29, 31]. This may be due to side effect of AEDs such as Phenobarbital as majority of our study participants used it.

Those patients who had difficulties of adherence to their AEDs had around five times (AOR = 4.80, CI 2.568, 8.964) odds of depression than those patients who adhere to their AEDs. The possible reason may be those patients who had difficulties of adherence to their AEDs may result in breakthrough seizures that may lead an individual to develop depression. Most studies show that uncontrolled seizures are associated with a higher prevalence of depression than seizure free patients [34]. Studies have shown a bidirectional interaction between epilepsy and depression with up to 60 % of epileptic patients develop depression and depression also increase risk of epilepsy up to 3–7 fold [16, 21]. Different studies reviled the relapse of seizures after discontinuation of AEDs and withdrawal of AEDs [35].

### Strength of the study

This study is the first of its kind in the study area to show the prevalence of depression among individual with epilepsy.

### Limitations of the study

This study has some important limitations that should be kept in mind when interpreting the results. First, the cross-sectional nature of the study design does not confirm definitive cause and effect relationship. Second the symptom similarity of depression and epilepsy, the adverse effects of AEDs overlap with symptoms of depression (such as fatigue, sleep disturbance, weight gain and memory problems) may over represent the problem. In addition, some of the independent variables like Seizure frequency, duration of illness and family history of mental illness relays on patient’s past history that might have recalled bias. Finally, some variables were assessed with single questions, for example, the difficulties of adherence to their AEDs that may lead some patients to respond improperly.

## Conclusion

Overall, the prevalence of depression was found to be high. Lower education status, early onset of illness, seizure frequency, poly-pharmacy and difficulties of adherence to AEDs were associated independently with depression. These findings add important evidence to the existing scant study in Sub-Saharan Africa and other developing countries on the psychological aspect of an individual with epilepsy. Stress reduction strategies (e.g., relaxation, exercise) particularly for those who had early onset of the illness and strengthening the educational status of the participants were suggested in the clinical care setting to enhance their adherence to AEDs and control seizure frequency. Additional researches with qualitative and quantitative study methods are also suggested, in order to explore the relation of socio-demographic and depression.
